# The Attenuation of Chronic Ulcerative Colitis by (R)-salbutamol in Repeated DSS-Induced Mice

**DOI:** 10.1155/2022/9318721

**Published:** 2022-02-07

**Authors:** Liangjun Deng, Haihua Guo, Shanping Wang, Xiaoming Liu, Yue Lin, Rui Zhang, Wen Tan

**Affiliations:** ^1^Institute of Biomedical and Pharmaceutical Sciences, Guangdong University of Technology, Guangzhou, 510006 Guangdong, China; ^2^Institute of Gastroenterology of Guangdong Province, Department of Gastroenterology, Nanfang Hospital, Southern Medical University, 510515 Guangzhou, China; ^3^Jeffrey Cheah School of Medicine and Health Sciences, Monash University Malaysia, Bandar Sunway 47500, Malaysia; ^4^Post-Doctoral Innovation Base, Jinan University Affiliation, Yuanzhi Health Technology Co., Ltd., Hengqin New District, Zhuhai, Guangdong 519000, China

## Abstract

Racemic salbutamol ((RS)-sal), which consist of the same amount of (R)-sal and (S)-sal, has been used for asthma and COPD due to its bronchodilation effect. However, the effect of (R)-sal on repeated dextran sulfate sodium (DSS)-induced chronic colitis has not yet been investigated. In this study evaluated the potential effect of (R)-, (S)-, and (RS)-sal in mice with repeated DSS-induced chronic colitis and investigated the underlying mechanisms. Here, we verified that chronic colitis was significantly attenuated by (R)-sal, which was evidenced by notably mitigated body weight loss, disease activity index (DAI), splenomegaly, colonic lengths shortening, and histopathological scores. (R)-sal treatment noticeably diminished the levels of inflammatory cytokines (such as TNF-*α*, IL-6, IL-1*β*, and IFN-*γ*). Notably, the efficacy of (R)-sal was better than that of (RS)-sal. Further research revealed that (R)-sal mitigated colonic CD4 leukocyte infiltration, decreased NF-*κ*B signaling pathway activation, improved the Nrf-2/HO-1 signaling pathway, and increased the expression of ZO-1 and occludin. In addition, (R)-sal suppressed the levels of TGF-*β*1, *α*-SMA, and collagen in mice with chronic colitis. Furthermore, the 16S rDNA sequences analyzed of the intestinal microbiome revealed that (R)-sal could mitigate the intestinal microbiome structure and made it more similar to the control group, which mainly by relieving the relative abundance of pathogens (such as Bacteroides) and increasing the relative abundance of probiotics (such as Akkermansia). Therefore, (R)-sal ameliorates repeated DSS-induced chronic colitis in mice by improving inflammation, suppressing oxidative stress, mitigating intestinal barrier function, relieving intestinal fibrosis, and regulating the intestinal microbiome community. These results indicate that (R)-sal maybe a novel treatment alternative for chronic colitis.

## 1. Introduction

Ulcerative colitis (UC) is one of the inflammatory bowel diseases (IBD) that is chronic and recurrent. The clinical characteristics of UC mainly include abdominal pain, weight loss, and bloody diarrhea [1–3], which seriously decrease the quality of life for patients [4]. The histopathological and inflammatory changes in UC are generally observed in the colon and rectum. UC is mainly prevalent in western countries in the past; however, in recent years, the incidences of UC have rapidly increased in Asia, such as China, India, and South Korea [5–8]. Although the exact pathogenesis of UC remains poorly understood, the accumulating data have suggested that UC is closely related to inflammation and oxidative stress imbalance, impairment of the intestinal barrier, and abnormalities in the intestinal microbiota [9–12].

Nuclear factor-kappa B (NF-*κ*B) is an important transcriptional regulation factor in the process of inflammation, usually in an inactive state due to its combination with its inhibitor kappa B (IKB) [13]. NF-*κ*B can be activated by stimulating factors, then induce the expression of a variety of genes, produce multiple cytokines to participate in the inflammatory response, and play an important role in maintaining normal physiological functions. However, when NF-*κ*B is overactivated, it will induce autoimmune diseases such as UC [13].

Nuclear factor erythroid 2-related factor 2 (Nrf-2) is an important transcription factor for antioxidative stress. Under normal conditions, Nrf-2 is linked to Kelch-Like ECH-Associated Protein 1 (Keap1) through E3 ubiquitin ligase and is degraded through ubiquitination. However, under oxidative stress, which can activate Nrf-2, then made it dissociates from Keap1, enters the nucleus, and upregulates the transcription of downstream antioxidant enzymes [14]. Thus, Nrf-2 can play a core role in antioxidative stress. It has been demonstrated that the expression of Nrf-2 was decreased in the DSS-induced UC model [15], which suggests that drugs can activate Nrf-2 which was expected to be applied in the treatment of UC.

The treatment medicines for UC depend on mesalamine, glucocorticoids, and immunomodulatory agents. However, the long-term use of these drugs is limited due to potential side effects, safety, and costs. Therefore, the development of alternative drugs for the treatment of UC is urgently needed.

Racemic salbutamol ((RS)-sal) is a short-acting *β*2-adrenergic receptor agonist consisting of an equal mixture of (R)-sal and (S)-sal. (RS)-sal can effectively inhibit the release of allergenic substances such as histamine and prevent bronchospasm, which commonly can be used for the treatment of bronchial asthma and asthmatic bronchitis [16]. Besides, there are some evidences demonstrating that *β*2-adrenergic receptor agonists exhibit anti-inflammatory effects by regulating the immune system [17, 18], which may contribute to the therapeutic effects in some inflammatory diseases, such as UC. A previous study in our group demonstrated that (RS)-sal exerted anti-inflammatory activity properties. However, the effect of sal in dextran sulfate sodium (DSS)-induced chronic colitis has not been investigated.

In view of this, the aim of our research was to evaluate the potential amelioration effect of (R)-sal in mice with repeated DSS-induced chronic ulcerative colitis and to elucidate the mechanisms underlying its therapeutic effect. In addition, we explored the differences in the mitigated effects of (R)-, (S)-, and (RS)-sal on chronic ulcerative colitis.

## 2. Materials and Methods

### 2.1. Materials

(R)-sal, (S)-sal, and (RS)-sal were supplied by Dongguan Key-Pharma Biomedical Company (Guangdong, China). DSS (MW 36-50 kDa) was obtained from MP Biomedicals Co., Ltd. (California, USA). The positive drug 5-ASA was obtained from Shanghai Yuanye Bio-Technology Co., Ltd. (Shanghai, China). The reagent for detecting fecal occult blood was supplied by Baso Diagnostics, Inc. (Zhuhai, China). The enzyme-linked immunosorbent assay (ELISA) kits for detecting the inflammatory cytokines interferon-*γ* (IFN-*γ*), interleukin- (IL-) 1*β*, IL-6, and tumor necrosis factor-*α* (TNF-*α*) were offered by Neobioscience Technology Company (Shenzhen, China). The antibodies of *β*-actin (ab227387), occludin (ab216327), and CD4 (ab183685) were obtained from Abcam Inc. (Burlingame, USA). The antibodies of zonula occludens-1 (ZO-1) (bs-1329R) and Nrf-2 (bs-1074R) were obtained from Bioss (Beijing, China). The antibodies of NF-*κ*B p65 (10745-1-AP) and heme oxygenase-1 (HO-1) (10701-1-AP) were purchased from Proteintech (Rosemont, USA). The antibody of phospho-NF-*κ*B p65 (p-NF-*κ*B p65) was purchased from Cell Signaling Technology (Danvers, USA). The antibodies of transforming growth factor-*β*1 (TGF-*β*1) (BA0290) and alpha smooth muscle actin (*α*-SMA) (BM0002) were offered by Boster Biological Technology Co., Ltd. (Wuhan, China). All chemical reagents employed were of analytical grade.

### 2.2. Animals

Male C57BL/6 mice (18-20 g, 6 weeks) were procured from the Medical Experimental Animal Centre of Southern Medical University (Guangzhou, China) and then housed under a pathogen-free experimental facility. These mice were acclimatized for seven days before being randomly divided into different experimental groups. All animal experimental protocols and care in this study were performed in compliance with the guidelines of the Institutional Animal Care and Use Committee and were approved by the Animal Experimentation Committee of Southern Medical University (L2017010).

### 2.3. Repeated DSS-Induced Chronic Colitis in Mice and Treated with (R)-, (S)-, and (RS)-sal

**Figure 1:**
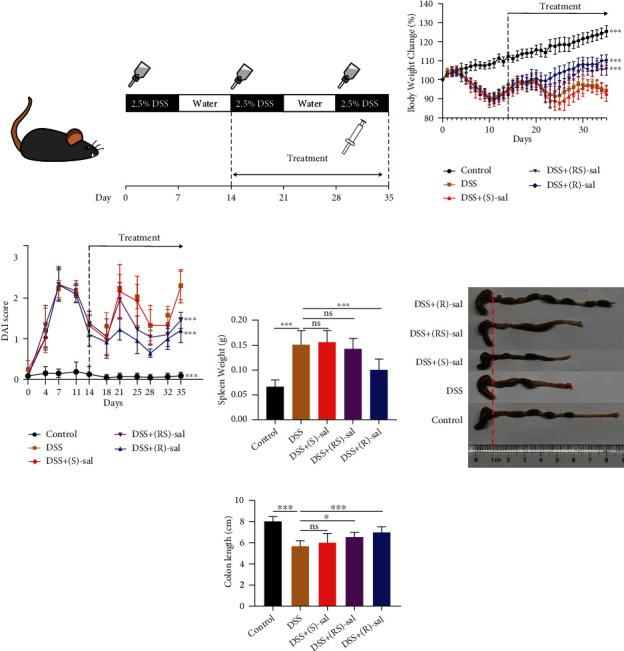
Effects of (R)-, (S)-, and (RS)-sal on repeated DSS-induced chronic colitis. (a) Schematic diagram of DSS-induced chronic colitis experiments. (b) Body weight change was recorded during the experiment (treatment from day 14 to day 35). (c) DAI scores were recorded at certain time points (treatment from day 14 to day 35). (d) Spleen weight in different groups. (e) Representative colons from different groups. (f) Colon length in different groups. ^∗^*P* < 0.05, ^∗∗^*P* < 0.01, ^∗∗∗^*P* < 0.001 versus the DSS group.

The mice were adapted to their environment for seven days, then randomly divided into 5 groups: the control group, DSS group, DSS+(S)-sal group (1.0 mg/kg), DSS+(RS)-sal group (2.0 mg/kg), and DSS+(R)-sal group (1.0 mg/kg) (*n* = 8). Experimental chronic colitis was induced by giving three cycles of 2.5% DSS according to the previous description [19, 20] with a slight modification. The first day of the experiment was defined as day 0. The timelines of this animal experiment are described in Figure 1(a). In brief, the control group only received water during the experiment. The other groups were induced by drinking 2.5% DSS treatment (three cycles for 7 days) and recovery by drinking water without DSS (two cycles for 7 days). The DSS+(S)-sal group, DSS+(RS)-sal group, and DSS+(R)-sal group were orally administered the corresponding drugs from day 14. The control group and DSS group were treated with the same dose of water. The body weight change in each group was detected once a day, and the disease activity index (DAI) was assessed at a specific time during the treatment period. DAI scores were blindly evaluated as described previously [21]. At day 35, mice were euthanized, and their blood samples were collected from the orbit. The colon samples were quickly removed, and their lengths were recorded. Subsequently, the colons were analyzed by histology examination. Spleen samples were quickly removed, and their weight was recorded.

### 2.4. Pharmaceutical Effect of (R)-sal on Repeated DSS-Stimulated Chronic Colitis

**Figure 2:**
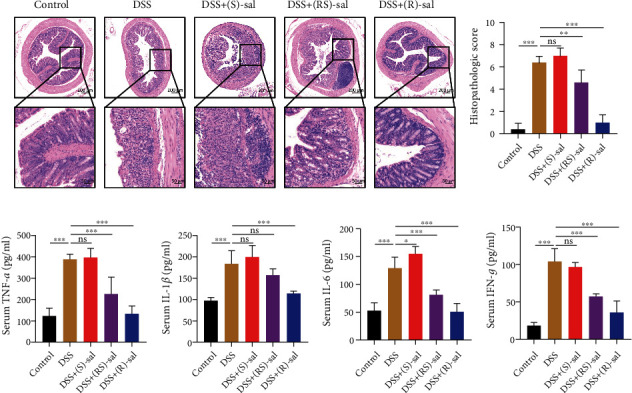
The mitigating effect of (R)-, (S)-, and (RS)-sal in the histological evaluation and the secretion of inflammatory cytokines. (a) Representative image of HE staining in different groups. (b) Histological scores of different groups. (c) TNF-*α*, (d) IL-1*β*, (e) IL-6, and (f) IFN-*γ* levels were measured by ELISA kits. ^∗^*P* < 0.05, ^∗∗^*P* < 0.01, ^∗∗∗^*P* < 0.001 compared with the DSS group.

The mice were adapted for seven days and then randomly divided into 5 groups: the control group, DSS group, DSS+5-ASA group (50 mg/kg), DSS+(R)-sal-L group (0.5 mg/kg), and DSS+(R)-sal-H group (1.0 mg/kg) (*n* = 8). The experimental chronic colitis was induced by giving three cycles of 2.5% DSS according to the previous description [19, 20] with slight modification. The first day of the experiment was defined as day 0. The timelines of this animal experiment are described in Figure 2(a). In brief, the control group only received water during the experiment. The other groups were induced by drinking 2.5% DSS treatment (three cycles for 7 days), and recovery was induced by drinking water without DSS (two cycles for 7 days). The DSS+5-ASA group, DSS+(R)-sal-L group, and DSS+(R)-sal-H group were orally administered the corresponding drugs from day 14. The control group and DSS group were treated with the same dose of water. The body weight change of each group was detected once a day, and the DAI was assessed at a specific time during the treatment period. DAI scores were blindly evaluated as described previously [21]. At day 35, the mice were euthanized, and their blood samples were collected from the orbit. The colon samples were quickly removed, and their lengths were recorded. Subsequently, the colons were divided into two sections: one section was analyzed via histology examination and the other was analyzed in biochemical assays. Spleen samples were quickly removed, and their weight was recorded.

### 2.5. Histopathological Examinations

After the mice were euthanized, distal colon samples were detached and immediately fixed in 10% formalin for 48 h. Next, the distal colon samples were embedded in paraffin, cut into 4 mm sections, then stained with hematoxylin and eosin (HE), periodic acid-Schiff (PAS), and picrosirius red staining. The images of samples were acquired by microscope. The colon histopathological scores were determined according to previously described methods [21] with slight modification, which was performed in a blind fashion and evaluated as follows: inflammation (scores varied from 0 to 4) and epithelium (scores varied from 0 to 4).

### 2.6. Detection of Inflammatory Cytokines by ELISA

The serum was obtained from whole blood samples by centrifugation (3000 rpm, 15 minutes). The concentrations of IFN-*γ*, IL-1*β*, TNF-*α*, and IL-6 in the serum were measured by respective ELISA kits based on the manufacturer's protocol.

### 2.7. Quantitative Real-Time PCR

Total RNA was isolated from colon samples using RNAprep pure Tissue Kit (TIANGE, Beijing, China) in RNase-free environment according to the manufacturer's protocol. RNA was reverse transcribed into cDNA by RevertAid First Strand cDNA Synthesis Kit (Thermo, MA, USA). The relative concentration of IFN-*γ*, IL-1*β*, TNF-*α*, and IL-6 was detected by FastStart Universal SYBR Green Master (Rox) (Roche, Basel, Switzerland). The primer sequences to detect IFN-*γ*, IL-1*β*, TNF-*α*, and IL-6 are revealed in Table 1. The expression of these mRNA was normalized with the reference gene GAPDH. Data were analyzed by 2^-*ΔΔ*CT^ method to evaluate the relative expression.

### 2.8. Western Blotting

The colon tissue protein was extracted by homogenization in radio immunoprecipitation assay (Biosharp, Anhui, China) and protease inhibitors (Biosharp, Anhui, China). The protein sample concentrations were measured by bicinchoninic acid assay kit (DINGGUO, Beijing, China) according to the manufacturer's instructions. After denaturation, the 10% sodium dodecyl sulfate-polyacrylamide gel electrophoresis was used to separate proteins. Then, the proteins were transferred onto polyvinylidene difluoride (PVDF) membranes. Each membrane was blocked by 5% skim milk for 1 h at room temperature. The membranes were exposed to the following primary antibodies overnight at 4°C with shaking: anti-*β*-actin (1 : 6000), anti-ZO-1 (1 : 1000), antioccludin (1 : 1000), anti-NF-*κ*B p65 (1 : 1000), anti-p-NF-*κ*B p65 (1 : 1000), anti-Nrf-2 (1 : 1000), and anti-HO-1 (1 : 3000). After that, each membrane was incubated with secondary antibody at 37°C for 1 h. Finally, the enhanced chemiluminescence reagent (BOSTER, Wuhan, China) was used to visualize each protein band. The protein bands were evaluated by ImageJ software, and *β*-actin was used to normalize the protein relate expression.

### 2.9. Immunofluorescence Measurement

Immunofluorescence was performed as described previously [22]. In brief, the paraffin-embedded colon tissues were sectioned, dewaxed, and rehydrated. Then, the slices blocked endogenous peroxidase activity with 3% H_2_O_2_, and antigens were repaired by citrate buffer solution. The slices were incubated with 3% bovine serum albumin to diminish nonspecific staining. The slices were incubated with the following primary antibodies at 4°C overnight: anti-CD4 (1 : 1000), anti-ZO-1 (1 : 500), antioccludin (1 : 200), anti-TGF-*β*1 (1 : 200), anti-*α*-SMA (1 : 200), and anti-Nrf-2 (1 : 400). After that, each slice was incubated with the respective secondary antibody for 30 min. Slices were counterstained with 4,6-diamidino-2-phenylindole (DAPI). The representative images were obtained under microscope, and the positive area of immunoreactivity was analyzed with Image-Pro Plus 6.0.

### 2.10. Gut Microbiota 16S rDNA Analysis

Gut contents (*n* = 8) were collected from the mice and stored at -80°C. DNA from the microbial community was extracted from the gut contents using the HiPure Soil DNA Kit. The quality and concentration of extracted DNA samples were evaluated by NanoDrop spectrophotometry (Thermo Scientific, Wilmington, USA). The V3-V4 variable region of the 16S rDNA gene was amplified by PCR. Subsequently, the PCR amplicons were purified by AMPure XP Beads (Beckman Agencourt, USA). Amplicons were extracted from 2% agarose gels and purified with AMPure XP Beads (Beckman Agencourt, USA), followed by quantification on the ABI StepOnePlus Real-Time PCR System (Life Technologies, Foster City, USA). The purified samples were sequenced and analyzed based on the Illumina platform. Sequencing service was provided by Genedenovo Inc. (Guangzhou, China).

### 2.11. Statistical Analysis

All data in these experiments were presented as the arithmetic mean ± standard deviation (SD). Statistical differences were analyzed by GraphPad Prism 8.0 (La Jolla, CA, USA). These results were analyzed by one-way or two-way ANOVA multiple comparison tests. *P* < 0.05, *P* < 0.01, and *P* < 0.001 were set as statistically significant.

## 3. Results

### 3.1. Differential Effects of (R)-, (S)-, and (RS)-sal on DSS-Induced Chronic Colitis

The colitis caused by DSS is similar to the pathological features of human colitis [20]. In order to assess the mitigation capacity of (R)-, (S)-, and (RS)-sal in chronic colitis, a chronic colitis mouse model was induced by DSS. The chronic colitis was induced by adding 2.5% DSS treatment in the drinking water (three cycles for 7 days), and recovery was by drinking water without DSS (two cycles for 7 days). (R)-, (S)-, and (RS)-sal were orally administered to mice from day 14 (Figure 1(a)). As exhibited in Figures 1(b) and 1(c), body weight loss and DAI scores were noticeably increased in repeated DSS-induced chronic colitis mice compared with the control group. (R)-sal and (RS)-sal dramatically relieved these changes compared with the DSS group. However, treatment with (S)-sal could not diminish the body weight loss or DAI scores. The colon length reduced, and splenomegaly was used to represent the severity of DSS-induced chronic colitis. As shown in Figure 1(d), oral administration of (R)-sal, instead of (S)-sal or (RS)-sal, notably suppressed spleen swelling compared with the DSS group. Furthermore, treatment with (R)-sal and (RS)-sal dramatically improved the colon length shortening compared with the DSS group (Figures 1(e) and 1(f)). However, (S)-sal treatment could not suppress this change in DSS-induced chronic colitis mice.

**Figure 3:**
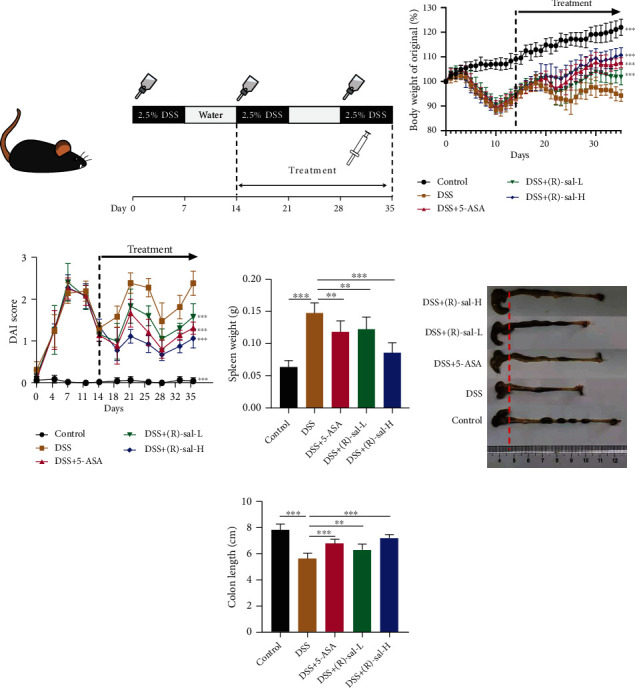
(R)-sal improved the symptoms on repeated DSS-induced chronic colitis. (a) Schematic diagram of repeated DSS-induced chronic colitis experiments. (b) Body weight change was measured daily during the experiment (treatment from day 14 to day 35). (c) DAI scores were recorded at certain time points (treatment from day 14 to day 35). (d) Spleen weight in different groups. (e) Representative colons from different groups. (f) Colon length in different groups. ^∗^*P* < 0.05, ^∗∗^*P* < 0.01, ^∗∗∗^*P* < 0.001 versus the DSS group.

Besides, the HE staining of colonic tissue was used to evaluate the histological effects of (R)-, (S)-, and (RS)-sal in repeated DSS-induced chronic colitis mice. As shown in Figures 3(a) and 3(b), compared with the control, the mice in repeated DSS-induced chronic colitis exhibited the intestinal barrier damage, reduced crypts, exacerbated inflammatory infiltration in the colon, and increased the histological score, whereas (R)-, (S)-, and (RS)-sal showed different effects on chronic colitis. (R)-sal and (RS)-sal dramatically suppressed inflammatory cell infiltration, intestinal barrier damage, and crypt destruction. Conversely, treatment with (S)-sal could improve nothing on chronic colitis.

It is well known that inflammatory cytokines play an important role in the pathogenesis of colitis. Therefore, in order to probe the anti-inflammatory effects of (R)-, (S)-, and (RS)-sal, the expressions of IFN-*γ*, IL-1*β*, TNF-*α*, and IL-6 were measured by ELISA. As shown in Figures 3(c)–3(f), these proinflammatory cytokines notably increased in repeated DSS-induced chronic colitis compared with the control group. Treatment with (R)-sal or (RS)-sal diminished the expression of these proinflammatory cytokines. Nevertheless, in comparison with the DSS group, administration of (S)-sal could not attenuate the production of proinflammatory cytokines. Collectively, these results demonstrated that (R)-sal should be further investigated for its attenuated effects on repeated DSS-induced chronic colitis.

### 3.2. (R)-sal Ameliorated the Symptoms on DSS-Induced Chronic Colitis

To evaluate the effect of (R)-sal on repeated DSS-induced chronic colitis, the mice with chronic colitis were treated with different doses of (R)-sal. 5-ASA was chosen as a positive control drug. This experiment scheme is illustrated in Figure 2(a). The body weight changes, DAI, spleen weight, and colon length, which are representative symptoms in chronic colitis, were recorded in this research. As exhibited in Figures 2(b)–2(f), (R)-sal notably mitigated these symptoms in a dose-dependent manner compared with the DSS group. Interestingly, the (R)-sal-H group even showed more improvement than the 5-ASA group in the aforementioned indices.

**Figure 4:**
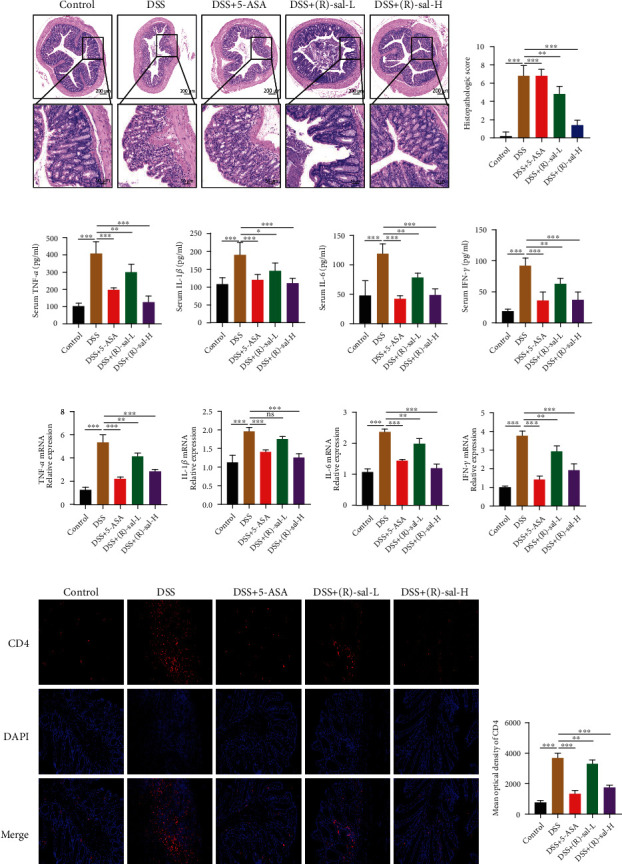
(R)-sal relieved the histological evaluation and inflammation on DSS-induced chronic colitis. (a) Representative image of HE in different groups. (b) Histological scores in different groups. (c) TNF-*α*, (d) IL-1*β*, (e) IL-6, and (f) IFN-*γ* levels were examined by ELISA kits. (g) TNF-*α*, (h) IL-1*β*, (i) IL-6, and (j) IFN-*γ* levels were examined by qPCR. (k) Representative image of CD4 staining in different groups. (l) The mean optical density of CD4 in different groups. ^∗^*P* < 0.05, ^∗∗^*P* < 0.01, and ^∗∗∗^*P* < 0.001 compared with the DSS group.

The histopathological examination of colon implied that DSS induced colon structure damage, the loss of crypt and goblet cells, and considerable inflammatory cell infiltration in the colon. As shown in Figures 4(a) and 4(b), the histological score of the DSS group was remarkably elevated compared with that of the control group, whereas the mice with repeated DSS-induced chronic colitis treated with different doses of (R)-sal had notably diminished damage. Compared with the DSS group, different doses of (R)-sal oral administration noticeably relieved the histological score in colon tissue. The result demonstrated that (R)-sal could outstandingly mitigate the colon injury in DSS-induced chronic colitis.

Moreover, inflammatory cytokines in the serum were investigated by ELISA. The results are shown in Figures 4(c)–4(f). The inflammatory cytokines, such as IFN-*γ*, IL-1*β*, TNF-*α*, and IL-6, were remarkably exacerbated in the DSS group compared with the control group, which indicated that inflammatory reactions were exacerbated in the DSS group. However, different doses of (R)-sal suppressed the expression of these inflammatory cytokines than the DSS group. Besides, the mRNA expression of these inflammatory cytokines in colon tissue was measured by qRT-PCR. As shown in Figures 4(g)–4(j), the mRNA expression of these inflammatory cytokines was noticeably exacerbated in the DSS group compared with the control group. In contrast, treatment with different doses of (R)-sal ameliorated the mRNA expression of inflammatory cytokines. These data indicate that (R)-sal exhibited an anti-inflammatory effect, which could inhibit the secretion of inflammatory cytokines on DSS-induced chronic colitis.

Sustained and chronic inflammation is one of the symptoms of chronic colitis and is closely related to its pathogenesis. CD4 leukocytes can induce inflammatory response from the immune system by diverse microbial pathogen activation. Therefore, to further analyze the improvement effects of (R)-sal in inflammation, we measured the expression of immune cell CD4 leukocytes in chronic colitis colon tissue by immunofluorescence. As shown in Figures 4(g) and 4(h), compared with the control group, CD4 leukocytes that infiltrated in colon structures were dramatically exacerbated, which means that the inflammatory response was activated in mice with chronic colitis. After (R)-sal oral treatment, the positive signal of CD4 was notably suppressed than the DSS group, which means that the inflammation was remarkably relieved.

### 3.3. (R)-sal Suppressed NF-*κ*B Signaling Pathway Activation in Mice with Chronic Colitis

**Figure 5:**
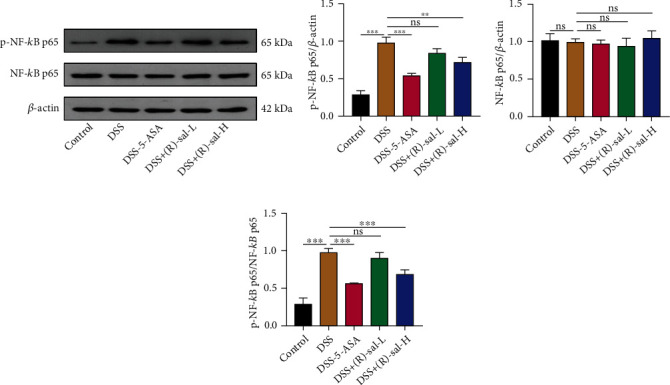
The effect of (R)-sal on NF-*κ*B pathway. (a) Western blot analysis of the expression of NF-*κ*B p65 and p-NF-*κ*B p65 in colon tissue. (b–d) The relative expression of p-NF-*κ*B p65, NF-*κ*B p65, and p-NF-*κ*B p65/NF-*κ*B p65, respectively. ^∗^*P* < 0.05, ^∗∗^*P* < 0.01, and ^∗∗∗^*P* < 0.001 versus the DSS group.

The NF-*κ*B signaling pathway induces the production of inflammatory factors in the pathological process of chronic colitis and plays core role in the inflammatory response. Consequently, the NF-*κ*B signaling pathway correlative proteins NF-*κ*B p65 and p-NF-*κ*B p65 were evaluated by western blot. As illustrated in Figure 5, the level of p-NF-*κ*B p65 in the DSS group was significantly increased than that in the control group, which means the NF-*κ*B signaling pathway was activated by induced DSS. Nevertheless, different doses of (R)-sal dramatically diminished the p-NF-*κ*B p65 expression in colon tissue compared with the DSS group. These results verified that (R)-sal suppressed NF-*κ*B pathway activation. Interestingly, different doses of (R)-sal exhibited little effect on NF-*κ*B p65 when compared with the DSS group.

### 3.4. (R)-sal Activated Nrf-2/HO-1 Expression in Mice with Chronic Colitis

**Figure 6:**
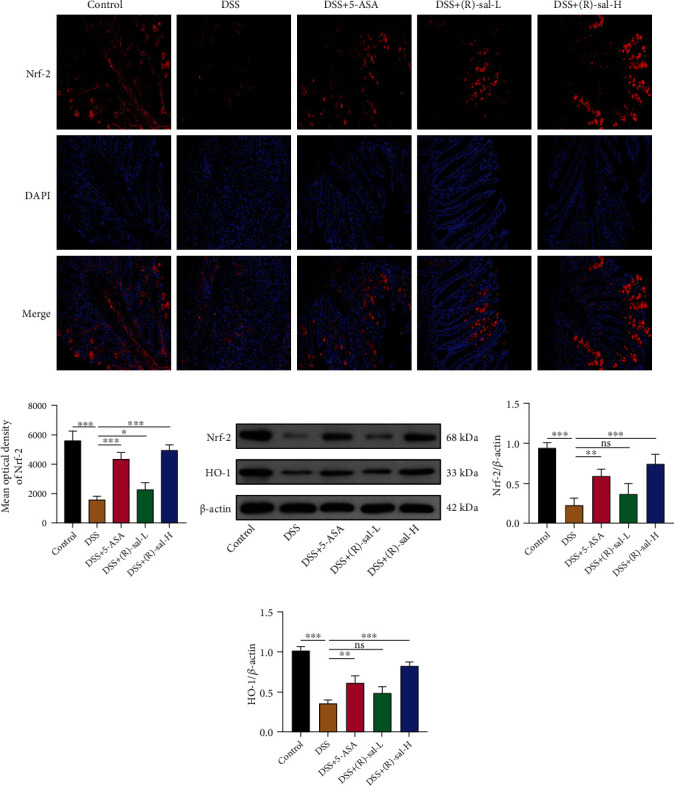
The effect of (R)-sal on Nrf-2/HO-1 pathway. (a) Representative image of Nrf-2 staining in different groups. (b) The mean optical density of Nrf-2 in different groups. (c) Western blot analysis of the expression of Nrf-2 and HO-1 in colon tissue. The relative expression of (d) Nrf-2 and (e) HO-1. ^∗^*P* < 0.05, ^∗∗^*P* < 0.01, and ^∗∗∗^*P* < 0.001 versus the DSS group.

The Nrf-2 signaling pathway exhibits antioxidative regulatory features. It has been reported that there was negative correlation between the Nrf-2 and the NF-*κ*B signaling pathway [23]. The oxidative environment leads to enlarging the level of ROS, which causes tissue damage and triggers inflammatory response [24, 25]. In order to evaluate the antioxidative role, the expression of Nrf-2 in the colon was detected by immunofluorescence. Compared with the control group, the dramatically attenuated Nrf-2 was detected in mice with chronic colitis, and this phenomenon was reversed by oral administration of (R)-sal in a dose-dependent manner (Figures 6(a) and 6(b)). Surprisingly, there was no noticeable variation in the (R)-sal-H group compared with the 5-ASA group.

To further assess the mechanism of (R)-sal, Nrf-2 and its downstream protein HO-1 were evaluated by western blot. As shown in Figures 6(c)–6(e), the expression of Nrf-2 and HO-1 were remarkably diminished in mice with chronic colitis, while treatment with different doses of (R)-sal dramatically elevated the levels of Nrf-2 and HO-1. Interestingly, the (R)-sal-H group exhibited upregulating the expression of Nrf-2 and HO-1 compared with the 5-ASA group. This is consistent with the above antioxidant results assessed by immunofluorescence, demonstrating that (R)-sal could notably activate the Nrf-2 signaling pathway.

### 3.5. (R)-sal Improved the Intestinal Barrier in Mice with Chronic Colitis

The intestinal barrier is a protection mechanism against damage from intestinal pernicious bacteria, which could prevent the exacerbation of colitis [26]. Glycogen protein, ZO-1, and occludin are crucial components of the intestinal barrier. Therefore, to observe the ameliorated effects of (R)-sal on intestinal barrier integrity, PAS staining and the expression of ZO-1 and occludin were analyzed by western blotting and immunofluorescence in the colon tissue samples.

**Figure 7:**
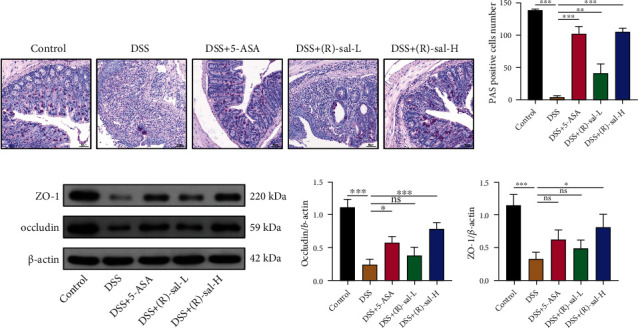
The effect of (R)-sal on intestinal barrier function. (a) Representative image of PAS staining from different groups. (b) The PAS positive cell number in different groups. (c) Western blot analysis of the expression of ZO-1 and occludin in colon tissue. The relative expression of (d) occludin and (e) ZO-1, respectively. ^∗^*P* < 0.05, ^∗∗^*P* < 0.01, ^∗∗∗^*P* < 0.001 versus the DSS group.

**Figure 8:**
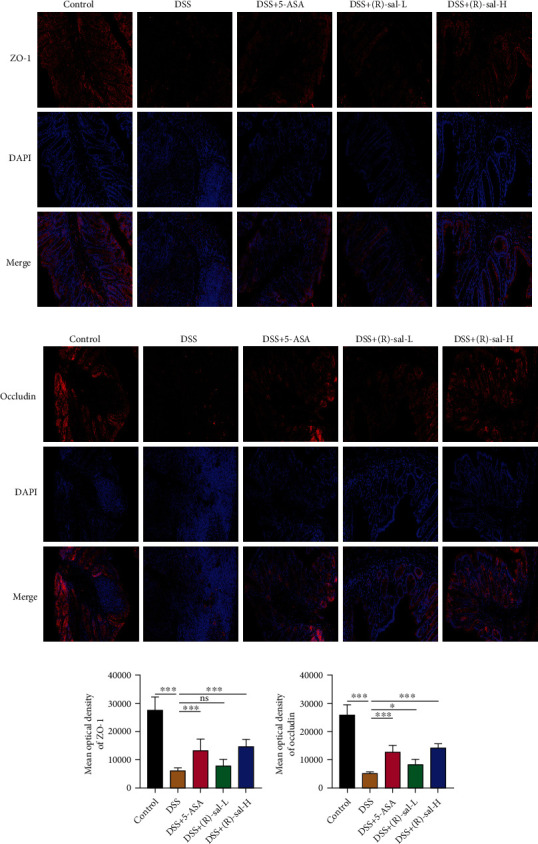
(R)-sal improved intestinal integrity by regulating the levels of ZO-1 and occludin. (a) Representative image of ZO-1 staining in different groups. (b) Representative image of occludin staining in different groups. (c) The mean optical density of ZO-1 in different groups. (d) The mean optical density of occludin in different groups. ^∗^*P* < 0.05, ^∗∗^*P* < 0.01, and ^∗∗∗^*P* < 0.001 versus the DSS group.

As shown in Figures 7(a) and 7(b), compared with the control group, goblet cells were dramatically damage in the DSS group. Oral administration of different doses of (R)-sal could notably elevate the number of goblet cells in the colon. Besides, in contrast to the control group, the expressions of ZO-1 and occludin were dramatically diminished in mice with chronic colitis, whereas (R)-sal reversed these changes in a dose-dependent fashion (Figures 7(c)–7(e)). Interestingly, the expressions of ZO-1 and occludin in the DSS+(R)-sal-H group were more elevated than those in the DSS+5-ASA group. Additionally, the expressions of ZO-1 and occludin were also investigated by immunofluorescence (Figure 8), and the results verified that (R)-sal could also upregulate the expression of ZO-1 and occludin in colon tissue, which was consistent with the western blot results. These results revealed that (R)-sal could improve the intestinal barrier in mice with chronic colitis.

### 3.6. (R)-sal Attenuated Intestinal Fibrosis in Mice with Chronic Colitis

**Figure 9:**
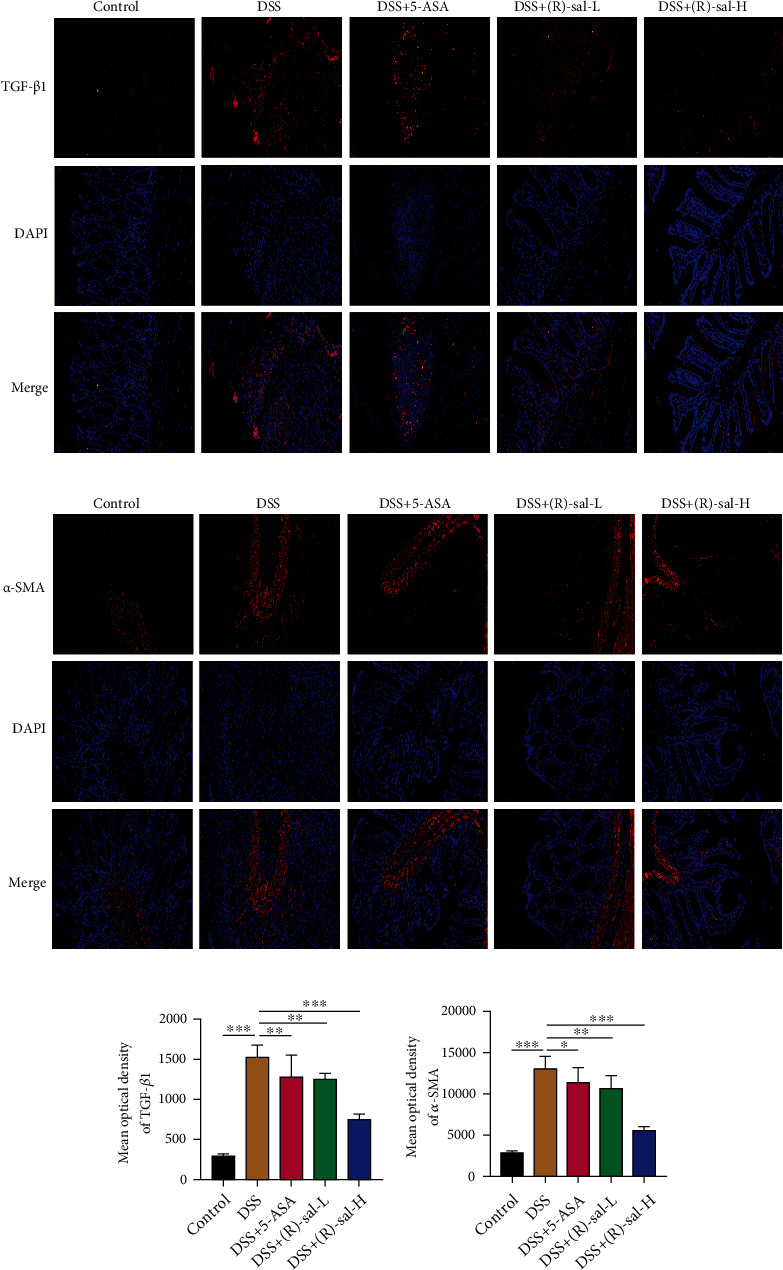
The effect of (R)-sal on intestinal tissue fibrosis. (a) Representative image of TGF-*β*1 staining in different groups. (b) Representative image of *α*-SMA staining in different groups. (c) The mean optical density of TGF-*β*1 in different groups. (d) The mean optical density of *α*-SMA in different groups. ^∗^*P* < 0.05, ^∗∗^*P* < 0.01, and ^∗∗∗^*P* < 0.001 versus the DSS group.

During the progression of chronic colitis, irregular myofibroblast activation results in extracellular matrix accumulation, eventually causing intestinal fibrosis [27]. TGF-*β*1 and *α*-SMA are the key biomarkers for intestinal fibrosis formation. Therefore, the effect of (R)-sal on intestinal fibrosis was assessed by TGF-*β*1 and *α*-SMA immunofluorescence. As shown in Figure 9, the results verified that there were lots of positive signals for TGF-*β*1 and *α*-SMA in colon tissue in the DSS group compared with the control group. These results demonstrated that intestinal fibrosis was detected in DSS-induced chronic colitis mice. Interestingly, compared with the DSS group, treatment with different doses of (R)-sal exhibited noticeable attenuation of the expression of TGF-*β*1 and *α*-SMA.

**Figure 10:**
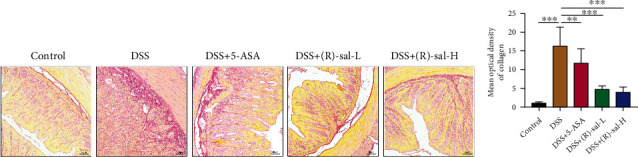
The effect of (R)-sal on the depositions of collagen. (a) Representative image of picrosirius red staining in different groups. (b) The mean optical density of collagen in different groups. ^∗^*P* < 0.05, ^∗∗^*P* < 0.01, and ^∗∗∗^*P* < 0.001 versus the DSS group.

Besides, the fibrosis-related collagen depositions in colon tissue were examined by picrosirius red stain. As shown in Figure 10, there were lots of collagen depositions on repeated DSS-induced chronic colitis mice compared with those on the control group. Importantly, oral administration with (R)-sal could appear to have improvement effects against collagen depositions, which contributed to suppressed intestinal fibrosis on DSS-induced chronic colitis mice. Clearly, the efficacy of the (R)-sal-H group was better than that of the 5-ASA group.

### 3.7. (R)-sal Regulated the Intestinal Microbiome in Mice with Chronic Colitis

Many researches have confirmed that the intestinal microbiome plays core role in UC pathogenesis [28, 29]. The modulation of the intestinal microbiome serves as a potential therapeutic strategy for chronic colitis. To further evaluate the effects of (R)-sal on the chronic colitis intestinal microbiome, the 16S rDNA sequencing of intestinal contents was performed.

**Figure 11:**
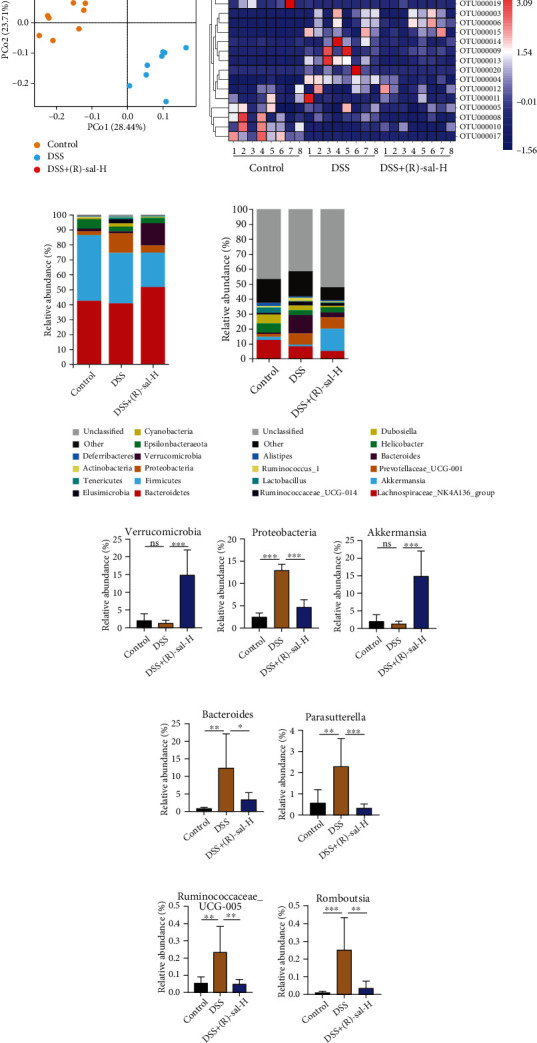
The effect of (R)-sal on the intestinal microbiome. (a) PCoA plot based on the weighted UniFrac index at the OTU level. (b) Heat map revealing the top 20 abundance microbial at the OTU level. (c) The intestinal microbiome structure at the phylum level. (d) The intestinal microbiome structure at the genus level. (R)-sal regulated (e) Verrucomicrobia and (f) Proteobacteria at the phylum level. (R)-sal modulated (g) Akkermansia, (h) Bacteroides, (i) Parasutterella, (j) Ruminococcaceae_UCG-005, and (k) Romboutsia at the genus level. ^∗^*P* < 0.05, ^∗∗^*P* < 0.01, and ^∗∗∗^*P* < 0.001 versus the DSS group.

Principal coordinate analysis (PCoA) based on the weight UniFrac distance matrices was used to observe the effect of (R)-sal on intestinal microbiome structural alterations. As shown in Figure 11(a), the intestinal microbiome was changed in the DSS group, which was notably different with the control group. However, oral treatment with (R)-sal changed the abnormal intestinal microbiome structure compared with the DSS group, making it more similar to the control group.

Besides, the heat map of OTU levels in the intestinal microbiome was used to further investigate the intestinal microbiome structure change in each group. The heat map exhibited the top 20 relative abundance OTU levels in each group. As shown by the heat map (Figure 11(b)), the intestinal microbiome structures among the control and DSS+(R)-sal-H groups were different than those among the DSS group, and the intestinal microbiome structure in the control group was similar to that in the DSS+(R)-sal-H group. The results of this analysis revealed that treatment with (R)-sal could diminish chronic colitis-induced changes in the intestinal microbiome structure at the OTU level.

In order to evaluate the effect of (R)-sal on adjusting the intestinal microbiome structure, the intestinal microbiome distribution at the phylum and genus levels was further investigated among these groups. As shown in Figure 11(c), the histograms exhibited species changes in the three groups at the phylum level and their relative abundance. Bacteroidetes, Firmicutes, and Proteobacteria were the preponderant species among these groups. The indicator value analysis (IndVal) was used to find the species with statistical significance. Verrucomicrobia and Proteobacteria were notably different species in each group (Figures 11(e) and 11(f)). Compared with the control group, the relative abundance of Verrucomicrobia was suppressed, and the relative abundance of Proteobacteria was elevated in the DSS group. Conversely, treatment with (R)-sal could change this trend.

Figure 11(d) illustrates alterations in the relative abundance of each group at the genus level. Compared with the control group, the relative abundances of Bacteroides, Parasutterella, Ruminococcaceae_UCG-005, and Romboutsia were raised to different degrees, and the relative abundance of Akkermansia was attenuated by DSS induction (Figures 11(g)–11(k)). However, these changes in bacteria could be ameliorated by the oral administration of (R)-sal. These results demonstrate that oral administration of (R)-sal could improve intestinal microbiome structure changes in mice with chronic colitis.

### 3.8. Safety Evaluation of (R)-sal

**Figure 12:**
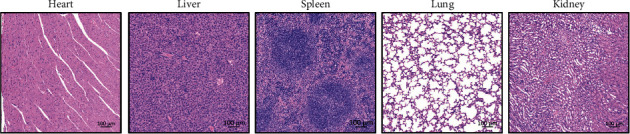
No toxicity was observed with the treatment of (R)-sal in mice with chronic colitis. Representative image of HE staining in the heart, liver, spleen, lung, and kidney tissue.

The safety of (R)-sal after long-term treatment in mice with chronic colitis was further investigated. There were no effects on the survival status of mice treated with (R)-sal during the study. The heart, liver, spleen, lung, and kidney tissues from the DSS+(R)-sal-H group were subjected to histopathological examination (Figure 12). The result verified that there was little notable damage in these tissues by oral administration of (R)-sal.

## 4. Discussion

UC is an inflammatory disorder that is characterized by recurrence and complicated etiology. Several factors play a core role in the pathogenesis of UC, including the environment, genetics, immune system, intestinal barrier, and intestinal microbiome [30, 31]. The potential adverse effects have limited current therapeutic drug applications [32, 33]. Therefore, it is necessary to develop safer and effective medicine for UC. In this study, we verified that (R)-sal, instead of (S)-sal or (RS)-sal, mitigated chronic colitis, with underlying mechanisms including the reduction in inflammatory reaction activation, activation of Nrf-2/HO-1 expression, improvement in the intestinal barrier, attenuation of intestinal fibrosis, and regulation of the intestinal microbiome in mice with chronic colitis.

It has been reported that there were different effects with regard to (R)-enantiomers and (S)-enantiomers in trials [34]. Therefore, different enantiomers were used to investigate the amelioration in chronic colitis. The results verified that (R)-sal, instead of (S)-sal, could noticeably improve chronic colitis in mice. In addition, although (RS)-sal contains the same amount of (R)-sal, the relieving effect of (RS)-sal on chronic colitis is less than that of single (R)-sal, which may be due to the existence of toxicity (S)-sal in (RS)-sal, weakening its amelioration effect. Therefore, there was significance beneficial effect to use (R)-sal in mice with chronic colitis.

Among the immune-regulatory factors, the inflammatory reaction has been thought to be a core mechanism in the pathophysiology of chronic colitis [35]. Proinflammatory cytokines play an active role in inflammatory reactions, which could induce macrophage migration and inflammatory mediator release [36], thereby further amplifying the inflammatory reaction. Previous studies have shown that proinflammatory cytokines were the typical features on repeated DSS-induced chronic colitis [37]. In this research, oral administration of (R)-sal dramatically suppressed the oversecretion of proinflammatory cytokines in mice with chronic colitis, which demonstrated that (R)-sal diminished abnormal inflammatory reactions. These results indicated that the colon exhibited an inflammatory state on repeated DSS-induced chronic colitis and that (R)-sal could improve this state. In addition, use of (S)-sal leads to further deterioration of this disease. Therefore, in chronic colitis, long-term use of (RS)-sal could weaken the effect of (R)-sal. In this model, colitis was repeatedly induced, and (R)-sal was given after the colitis appeared in order to investigate the therapeutic effect. In addition, this study showed significant therapeutic effect of (R)-sal; furthermore, (R)-sal was more effective in either active or recovery states when colitis was induced by DSS or withdraw, in compassion of (RS)-sal and 5-ASA.

To further investigate the anti-inflammatory mechanism of (R)-sal, the NF-*κ* Brelated proteins NF-*κ*B p65 and p-NF-*κ*B p65 were detected in colon tissue. NF-*κ*B plays a core role in regulating the process of inflammation [38]. It has been reported that activation of NF-*κ*B could elevate proinflammatory cytokine expression [39]. These cytokines trigger positive feedback regulation in inflammation activation, which ultimately damages colon tissue [40, 41]. The results of this study revealed that the levels of p-NF-*κ*B p65 were dramatically increased in the DSS group compared with the control group, which illustrated that the NF-*κ*B signaling pathway was activated in mice with chronic colitis. Treatment with (R)-sal dramatically suppressed p-NF-*κ*B p65 expression, which was consistent with previous proinflammatory cytokine expression changes. These results demonstrated that (R)-sal could relieve inflammation by mitigating the activation of the NF-*κ*B pathway.

The Nrf-2 signaling pathway is a significant pathway which involved in regulating the level of antioxidant medium [42, 43]. The Nrf-2 downstream antioxidant protein HO-1 could increase expression after Nrf-2 pathway activation [44]. HO-1 is an antioxidant protein that constitutes a defense network against oxidative stress damage and prevents colon tissue oxidative damage [45, 46]. Besides, there is an interaction between Nrf-2 and NF-*κ*B pathway [47]. Suppression of the expression of Nrf-2 would exacerbate oxidative stress generation, which further induces NF-*κ*B activation. Furthermore, CREB binding protein (CBP) is a transcription factor between Nrf-2 and NF-*κ*B, which means that there is competition between Nrf-2 and NF-*κ*B, namely, negative feedback regulation [48]. Saber et al. [49] found that olmesartan could notably improve colitis due to olmesartan being able to suppress NF-*κ*B and activate Nrf-2. In this research, the expression of Nrf-2 and HO-1 in colon tissue was remarkably relieved in mice with chronic colitis compared with control mice, and this abnormality could be noticeably reversed by oral administration of (R)-sal. These results demonstrated that (R)-sal could relieve chronic colitis by activating the Nrf-2 pathway.

The intestinal barrier protects against the invasion of pathogenic microorganisms and diminishes colon tissue damage [50, 51]. Tight junction proteins (such as the cytoplasmic scaffolding protein ZO-1 and the transmembrane barrier protein occludin) and glycogen proteins are core elements in the intestinal barrier, which play crucial roles in maintaining the integrity of the intestine [52]. Damage to the intestinal barrier is considered one of the core factors for colitis formation [53]. Therefore, the protective effects of (R)-sal in PAS, ZO-1, and occludin were further investigated in mice with chronic colitis. Similar to previous researches, the expressions of ZO-1, occludin, and glycogen proteins were noticeably suppressed compared with the control group, which reflected that the intestinal barrier was damaged in mice with chronic colitis. Surprisingly, treatment with (R)-sal remarkably elevated the levels of ZO-1, occludin, and glycogen proteins, which improved the integrity in colon tissue. These results demonstrated the improved effect of (R)-sal on intestinal barrier integrity, which may offer potential therapy for chronic colitis.

Intestinal fibrosis is considered a crucial element in the evolution of chronic colitis [54]. Chronic inflammation continuously stimulates related cell proliferation and persistent depositions of collagen, which caused extracellular matrix (ECM) abnormally exacerbates and sedimentary, eventually leading to intestinal fibrosis [55]. Mesenchymal cells, epithelial cells, endothelial cells, stellate cells, and fibrocytes contribute to ECM accumulation [56, 57]. Previous research has demonstrated that lots of ECM accumulated in the colon tissue, which leads to the formation of colon fibrosis in IBD patients [58]. TGF-*β*1 is a key profibrotic cytokines which secreted by a variety of cells such as intestinal fibroblasts and intestinal epithelial cells in colon tissue [56]. During chronic colitis, the secretion of TGF-*β*1 is exacerbated, which acts on intestinal interstitial cells to elevate ECM secretion [59, 60]. Therefore, the expression of TGF-*β*1 could reflect the degree of intestinal fibrosis. These results demonstrated that the expression of TGF-*β*1 was dramatically elevated in the colon of mice with chronic colitis, and this abnormal performance was attenuated by oral administration of (R)-sal. In addition, the biomarker of fibrosis *α*-SMA and the deposits of collagen were further assessed in colon tissue. The upregulated level of *α*-SMA and collagen was proven in mice with chronic colitis, and treatment with (R)-sal reversed this status. Use of 5-ASA has less role in fibrosis development, while by surprise, (R)-sal showed a remarked protective effect in antifibrosis.

Recently, intestinal microbiome dysbiosis was thought to play the crucial role in activating the immune defense system [61–63]. Many researches have reported that intestinal microbiome dysbiosis was closely related to the progression of UC [40, 64]. Besides, according to many researches, treatment with DSS in mice could remarkably alter the intestinal microbiome composition [65, 66]. To further assess the effect of (R)-sal on the intestinal microbiome structure on DSS-induced chronic colitis mice, 16S rDNA gene sequencing was executed in different groups. The PCoA parameters were used to evaluate similarities in these groups. As expected, some changes in the microbiome structure were found in the DSS and (R)-sal-H groups. In this research, based on PCoA parameter analysis, the microbiome structure was more similar between the control and (R)-sal-H groups at the OTU level than the DSS group. Moreover, the heat map of microbial composition at the OTU level also further confirmed this phenomenon. These findings demonstrated that treatment with (R)-sal could ameliorate the intestinal microbiome structure, which was different from the DSS group.

To further investigate the improvement in microbial composition by (R)-sal, the gut microbiota structure composition at the taxonomic hierarchy level was examined. At the phylum level, Bacteroidetes, Firmicutes, and Proteobacteria were found to compose the main part of the microbiota structure, which is similar to previous research [67, 68]. The intestinal microbiome in mice with chronic colitis showed that the ratio of Proteobacteria was dramatically exacerbated, which was consistent with a previous study [40]. Oral administration of (R)-sal reversed the ratio of Proteobacteria in mice with chronic colitis. It has been reported that Proteobacteria was thought to be harmful [66]. Verrucomicrobia was thought to be closely related to regulation of the mucosal inflammation [69]. Emerging studies have shown the ratio of Verrucomicrobia was suppressed in UC patients [70]. Treatment with (R)-sal notably elevated the ratio of Verrucomicrobia, which helped to improve the mucosal inflammation in chronic colitis.

At the genus level, (R)-sal could also reverse some special microbial ratio changes in mice with chronic colitis. Treatment with DSS dramatically elevated the relative abundances of Bacteroides, Parasutterella, Romboutsia, and Ruminococcaceae_UCG-005; nevertheless, treatment with (R)-sal could regulate these changes in varying degrees. Bacteroides has been found to be noticeably elevated in UC patients, which could stimulate and aggravate colon inflammation [71, 72]. Parasutterella could induce enteritis and septicemia, and it has been reported that the relative abundances were positively correlated with intestinal chronic inflammation and irritable bowel syndrome severity [73–75]. Therefore, suppression of the level of proinflammatory microbiota could help to regulate inflammation, thus mitigating chronic colitis.

Akkermansia is a probiotic that can maintain intestinal barrier function and regulate the immune response by producing various metabolites [76]. It has been reported that the relative abundance of Akkermansia was notably attenuated in UC patients [77]. Surprisingly, (R)-sal remarkably elevated the relative abundance of Akkermansia compared with the mice with chronic colitis, which could contribute to improve the intestinal barrier function and regulate the immune response. In this research, treatment with (R)-sal was found to regulate the relative abundances of some special intestinal microbiomes, which helped to promote the amelioration of chronic colitis.

## 5. Conclusion

In conclusion, the results of our study demonstrate (R)-sal significantly effective in treatment chronic colitis. Furthermore, that (R)-sal, instead of (S)-sal or (RS)-sal, markedly attenuate chronic colitis in mice through several mechanisms, including suppressing gut inflammation, regulating oxidative stress, ameliorating intestinal barrier integrity, suppressing colon fibrosis, and diminishing intestinal microbiome dysbiosis. These results reveal a therapeutical potential of long-term usage of (R)-sal in chronic colitis with reduced side effect in comparison of other traditional therapeutical options.

## Figures and Tables

**Table 1:** The primers used in qPCR assay.

Gene	Primers	Sequences (5′-3′)
TNF-*α*	Forward	AAGTTCCCAAATGGCCTCCC
	Reverse	CCACTTGGTGGTTTGTGAGTG
IL-1*β*	Forward	GCAGTGGTTCGAGGCCTAAT
	Reverse	GCTGCTTCAGACACTTGCAC
IL-6	Forward	GACAAAGCCAGAGTCCTTCAGA
	Reverse	TGTGACTCCAGCTTATCTCTTGG
IFN-*γ*	Forward	AGACAATCAGGCCATCAGCAA
	Reverse	GTGGGTTGTTGACCTCAAACT
GAPDH	Forward	CCTCGTCCCGTAGACAAAATG
	Reverse	TGAGGTCAATGAAGGGGTCGT

## Data Availability

The data underlying this article are available in the article.
